# Pursuing a Curative Approach in Multiple Myeloma: A Review of New Therapeutic Strategies

**DOI:** 10.3390/cancers11122015

**Published:** 2019-12-13

**Authors:** Mattia D’Agostino, Luca Bertamini, Stefania Oliva, Mario Boccadoro, Francesca Gay

**Affiliations:** Myeloma Unit, Division of Hematology, University of Torino, Azienda Ospedaliero-Universitaria Città della Salute e della Scienza di Torino, 10126 Torino, Italy

**Keywords:** multiple myeloma (MM), newly diagnosed, smoldering, minimal residual disease (MRD), high risk, autologous stem-cell transplantation (ASCT)

## Abstract

Multiple myeloma (MM) is still considered an incurable hematologic cancer and, in the last decades, the treatment goal has been to obtain a long-lasting disease control. However, the recent availability of new effective drugs has led to unprecedented high-quality responses and prolonged progression-free survival and overall survival. The improvement of response rates has prompted the development of new, very sensitive methods to measure residual disease, even when monoclonal components become undetectable in patients’ serum and urine. Several scientific efforts have been made to develop reliable and validated techniques to measure minimal residual disease (MRD), both within and outside the bone marrow. With the newest multidrug combinations, a good proportion of MM patients can achieve MRD negativity. Long-lasting MRD negativity may prove to be a marker of “operational cure”, although the follow-up of the currently ongoing studies is still too short to draw conclusions. In this article, we focus on results obtained with new-generation multidrug combinations in the treatment of high-risk smoldering MM and newly diagnosed MM, including the potential role of MRD and MRD-driven treatment strategies in clinical trials, in order to optimize and individualize treatment.

## 1. Introduction

Multiple Myeloma (MM) is a hematologic malignancy caused by the outgrowth of monoclonal plasma cells that leads to end-organ damage [1]. In 2018, MM accounted for 1.2% of all cancer diagnoses and 1.6% of all cancer deaths in Europe [2]. The median overall survival (OS) of newly diagnosed (ND) MM patients improved from 3.9 years for patients diagnosed between 2004 and 2007 to 6.3 years for those diagnosed between 2008 and 2012 to a median OS that is not yet reached in patients diagnosed after 2012 [3]. The introduction of new drug classes like proteasome inhibitors (PIs), immunomodulatory drugs (IMiDs), and, more recently, monoclonal antibodies (mAbs) has been the main determinant of the observed OS improvement, together with an improved supportive care. Nevertheless, the main cause of death in MM patients is still the development of drug-resistant disease [4]. Although obtaining deep responses is a universally recognized predictive factor of good outcome [5], long-term disease control, rather than disease eradication, is still the aim of MM treatment in current clinical practice, since the available data show that even patients achieving minimal residual disease (MRD) negativity relapse. This confirms the so-far incurable nature of MM. Recent data, comparing the survival of young MM patients treated between 2005 and 2015 to that of young patients affected by curable hematologic diseases (e.g., diffuse large B cell lymphoma (DLBCL) and Hodgkin Lymphoma (HL)) [6] and to that of the general population showed that MM patients have a 20-fold excess mortality compared to the general population, while DLBCL and HL have a non-significant excess mortality [6].

However, more recent results from clinical trials exploring novel three-drug and four-drug combinations showed unprecedented rates of prolonged and deep responses [7,8,9,10,11], with acceptable safety profiles even in elderly patients, thus increasing the likelihood not only to achieve disease control, but potentially cure, at least in a subset of patients. To design a potentially curative strategy, we have to focus on the first stages of the disease (smoldering MM (SMM), newly diagnosed MM (NDMM)), when the patient is treatment-naïve and disease genomic complexity is lower, as compared to the advanced relapsed/refractory setting.

In this review, we provide a summary of the new techniques used to detect residual disease at high sensitivity and of the results obtained in SMM and NDMM with new-generation combinations. We also explore how we can exploit these data in the future, towards a potential cure of MM.

## 2. Evolution of Response Criteria and MRD Techniques 

To enable disease eradication strategies, it is mandatory to have sensitive methods to detect small amounts of residual disease after treatment. MM response criteria evolved together with therapies. While before the introduction of novel agents, the rates of complete remission (CR) were very low (2% after 3 cycles of vincristine–doxorubicin–dexamethasone [12]), with novel combinations CR can now be obtained in >60% of patients [9]. Conventionally, MM response is evaluated measuring M-protein levels in the blood and urine, but it is now clear that even when M-protein disappears, residual disease can still be present [13]. The Spanish group showed that, among patients with a conventionally defined CR, there was a significant difference in outcome between MRD-negative and MRD-positive patients (median progression-free survival (PFS) 63 vs. 27 months, *p* < 0.001; median OS not reached vs. 59 months, *p* < 0.001). Interestingly, the outcome of patients with MRD-positive CR was similar to the outcome of those achieving only a partial response (PR), thus suggesting that the advantage of reaching CR over PR relies on the MRD-negative status. Recently, response criteria have been updated, introducing a universal definition of MRD beyond CR (for a detailed definition of the updated response criteria, please refer to Kumar et al., 2016) [14,15,16,17]. 

Two techniques have been developed and validated to detect MRD into the bone marrow: multiparameter flow cytometry (MFC) and next-generation sequencing (NGS).

MFC detects and quantifies tumor plasma cells using cell surface and cytoplasmic markers. Neoplastic plasma cells are characterized by the aberrant expression of molecules like CD19, CD20, CD27, CD28, CD33, CD38, CD45, CD56, CD117, and surface membrane immunoglobulin [18]. The first attempts to detect MRD by MFC had a maximum sensitivity of 10^−4^–10^−5^. The optimization of the MFC assay using two 8-color tubes, a bulk-lysis procedure, the acquisition of ≥10^7^ cells/sample, and the automatic plasma cell gating through a software tool led to reproducible results and enhanced the maximum sensitivity to 10^−5^–10^−6^ (next-generation flow, NGF) [19,20]. Using NGF, Flores-Montero and colleagues demonstrated that 25% of patients who were classified as MRD-negative by second-generation MFC were indeed MRD-positive by NGF [20]. Moreover, NGF negativity predicted a significantly longer PFS than second-generation MFC negativity among CR patients (*p* = 0.02) [20].

NGS technique was mainly developed by Adaptive Biotechnologies (Seattle, WA, USA) by producing and validating ClonoSEQ® Assay, which has recently obtained, by the Food and Drug Administration (FDA), the authorization as standardized technique for the disease evaluation in MM patients [21]. In this test, DNA from the immunoglobulin genes is amplified and sequenced using baseline bone marrow sample and identical sequences detected in more than 5% of the reads are identified as clonal gene rearrangements. These rearrangements are then searched in follow-up samples to identify MRD [22,23]. NGS reaches maximum sensitivity up to 10^−6^ [21].

Ongoing clinical trials are evaluating NGS vs. NGF/MFC and their correlation [24], and will help understand if the two techniques can be considered equivalent in identifying MRD negativity at a specific cut-off. Each technique has its own advantages and drawbacks (Table 1).

The maximum sensitivity reached is a key point, especially in a curative perspective. Each log depletion in MRD levels predicts a 1-year median OS advantage (5.9 years at 10^−2^–10^−3^, 6.8 years at 10^−3^–10^−4^, and ≥7.5 years at 10^−4^), suggesting that MRD levels at the highest sensitivity should be pursued [25]. Several reports suggested that once MRD-negative statuses are reached with a high sensitivity technique, patient prognoses are similar independently from the treatment that induced MRD negativity [26,27]. This observation also seems to apply to patients with adverse baseline prognostic factors (e.g., high-risk cytogenetics or elevated Revised International Staging System (R-ISS) stage), among whom MRD-negative patients at a sensitivity of 10^−5^–10^−6^ [26,28], but not at a sensitivity of 10^−4^ [29], showed similar clinical outcomes compared to standard-risk patients. Nevertheless, reaching MRD negativity in high-risk patients may be harder [30], and intensive regimens are likely needed in this patient population [8,9].

Even when evaluating MRD at a sensitivity of 10^−6^, there are still patients that can relapse. Relapses can also be caused by extramedullary disease [31]. Fluorodeoxyglucose (FDG) positron emission tomography/computed tomography (PET/CT) is currently considered the standard of care to assess residual disease outside the bone marrow [32,33]. The predictive role of post-treatment PET/CT has been demonstrated by different studies [34,35,36] and, in a head-to-head comparison [34], the normalization of PET/CT outperformed that of conventional magnetic resonance imaging after therapy for the prediction of PFS and OS. Recently, Zamagni et al. presented data on the standardization of PET/CT to define criteria for MRD negativity using the 5-point Deauville score. PET/CT imaging was a reliable predictor of outcomes regardless of treatment. The achievement of a Deauville score ≤3 was the predictor of a longer time to disease progression and overall survival (OS) and, consequently, a potential standardized definition of PET/CT negativity [37].

MRD assessed by PET/CT and bone marrow techniques synergistically predict patient outcome, with the best PFS detected in patients who were MRD-negative both within and outside the bone marrow [38]; hence the definition of Imaging MRD negativity.

Both the NGS and the NGF-plus-Imaging approaches are needed for the response evaluation in the setting of a curative strategy. Nevertheless, from a practical perspective, it should be determined if all these techniques are necessary for all patients, or if it is possible to develop an algorithm to define how to proceed. To do this, we need to answer open questions such as: In which patients should we perform MRD (CR, stringent CR (sCR) very good partial response (VGPR))? What is the proportion of patients who are still PET-positive despite being MRD-negative in the bone marrow with a high cut-off level (e.g., 10^−6^)? Vice versa, how many PET-positive patients are MRD-negative? Who are the patients that show discrepancies between the two evaluations? Ongoing studies including both BM and PET/CT evaluation at specific time points will help in drawing conclusions [37]. 

In the future, liquid biopsy approaches that use peripheral blood samples could potentially overcome the need to assess MRD both in the bone marrow and by imaging. However, these techniques are still at early developmental stages [39]. 

Besides achieving MRD negativity, a more important factor is maintaining it [40]: here comes the definition of “sustained MRD negativity” by the International Myeloma Working Group (IMWG), which uses the 1-year cut-off. An effort should be made to define the optimal duration of MRD negativity to reach an “operational cure”; this still remains an unanswered question, with a potentially great clinical relevance. For instance, in the chronic myeloid leukemia field, a sustained major molecular response lasting at least 2 years is usually required to be a candidate for treatment discontinuation [41], and longer deep molecular response durations prior to discontinuation are associated with the increasing probability of maintaining a major molecular response after discontinuation [42]. Little data are available in MM. Using MFC to monitor MM patients after induction and at different time points post-autologous stem-cell transplantation (ASCT), Gu and colleagues showed that, among patients achieving MRD negativity after the post-induction time point, MRD reappearance can happen 18–24 months after ASCT, thus suggesting that long-term confirmation of sustained MRD negativity may be necessary [43].

## 3. Treatment of High-Risk SMM

SMM [44] is an asymptomatic plasma cell neoplasm harboring a variable risk of progression to MM. Several scores have been proposed to assess SMM risk of progression to symptomatic MM (Table 2) [45,46]. 

The 2/20/20 model was the most recently proposed; its name comes from the resulting cut-offs of M-protein, bone marrow plasma cells (BMPC), and free light chains (FLC). M-protein >2g/dL (hazard ratio (HR) 1.56, *p* = 0.01; BMPC % >20% (HR 2.28, *p* < 0.0001), and FLC ratio (FLCr) >20 (HR 2.13, *p* < 0.0001)) independently predicted shorter time to progression (TTP) in multivariate analysis. Three risk groups were identified: Low risk (none of the risk factors), intermediate risk (1 risk factor), and high risk (≥2 risk factors), with a median TTP of 110, 68, and 29 months, respectively (*p* < 0.0001) [45]. The high-risk group consisted of 36% of the analyzed cohort of SMM.

A retrospective multicenter study by the IMWG validated the 2/20/20 model; furthermore, incorporating the cytogenetic abnormalities detected by fluorescence in situ hybridization (FISH; presence vs. absence of t(4,14), t(14,16), 1q gain, and/or del13), they identified four risk categories with a 2-year progression risk of 3.7% (low risk), 25% (low–intermediate), 49% (intermediate–high), and 72% (high) [49].

The current standard of care for SMM is periodical monitoring, with a suggested frequency based on patient risk to identify the possible evolution to symptomatic MM in due time and avoid severe organ damage. While this strategy suits well low-risk SMM patients who are unlikely to progress to MM, it may be questionable in high-risk SMM. In this setting, open questions are: (1) Can these patients benefit from an early therapy aiming at delaying the very likely evolution to MM? (2) Is there a possibility that early treatment may actually cure the disease? The latter hypothesis is based upon the very good outcome observed in patients with symptomatic MM and good prognosis [50], as well as upon a lower genomic complexity during the early phases of the disease that, together with a lower tumor burden, might suggest a higher possibility of cure [51]. Moreover, better treatment feasibility is expected in asymptomatic patients in good conditions. This rationale led to the design of clinical trials for the treatment of high-risk SMM (Table 3).

In the phase III randomized QuiRedex study, 119 high-risk SMM patients [52] received lenalidomide–dexamethasone (Rd) vs. observation. After a median follow-up of 75 months, the median TTP was not reached in the Rd group (*n* = 57) vs. 23 months in the observation group (*n* = 62, HR 0.24, *p* < 0.0001). An advantage in OS in the Rd arm was also detected (HR 0.43, *p* = 0.024). Interestingly, survival was similar in the two groups for patients who had previously received subsequent lines of therapy at the progression to active disease (HR 1.34, *p* = 0.50). The Rd combination showed acceptable levels of toxicity: Grade ≥3 adverse events (AEs) were infection (6%), asthenia (6%), neutropenia (5%), and skin rash (3%). During treatment, two patients treated with lenalidomide died of infection. A higher rate of second primary malignancies (SPMs) was detected in the Rd group (10%) vs. the observation group (2%). Of note, progression was defined by classical CRAB criteria (hyperCalcemia, Renal failure, Anemia, and Bone lesions) and advanced imaging techniques at screening were not performed, thus suggesting that the study also included patients who would currently be classified as having symptomatic MM. 

The efficacy of lenalidomide was also shown in the phase II/III E3A06 study, in which lenalidomide was compared to observation in SMM [62]. After a median follow-up of 35 months in phase III of the trial, the overall response rate (ORR) was 50% in the R group and 0% in the observational group. One-year, 2-year, and 3-year PFS were respectively 98%, 93%, and 91% in the R group, favorably comparing with respectively 89%, 76%, and 66% in the observational arm (HR 0.28, *p* = 0.002). Among lenalidomide-treated patients, grade 3/4 non-hematologic toxicities occurred in 28% of the phase III patients, with hypertension and infections being most common toxicities. However, no difference in scores regarding the quality of life was noted between the lenalidomide and the observational groups. In this trial, SPMs were detected in 11.4% of lenalidomide-treated patients vs. 3.4% of patients in the observational group.

In this setting, another attractive drug is the second-generation PI ixazomib, which is characterized by a convenient oral administration and shows good safety results. In a phase I study, ixazomib associated with dexamethasone showed good tolerability and high response rate (ORR 64%, PR 57%, and VGPR 14%) [55]. A phase II study exploring the entirely oral triplet ixazomib–lenalidomide–dexamethasone confirmed the good tolerability profile and efficacy of this combination, with a 58% of ≥VGPR (CR 19%, VGPR 34%) [58].

MAbs were also evaluated for the treatment of SMM. The anti-SLAMF7 elotuzumab as single agent showed a low response rate (ORR 10%, minimal response (MR) 29%), with a 2-year PFS of 69%, while first data of the combination with Rd showed a ≥VGPR rate of 43% [56,57].

The phase II CENTAURUS trial evaluated daratumumab alone in 123 patients with three different dose schedules and durations (long intense, intermediate, short intense; Table 3) [60]. At a median follow-up of 25.9 months, ≥VGPR rates were higher in the long intense and intermediate arms compared to the short intense arm (29%, 25%, and 18%, respectively). The 24-month PFS rates were 90%, 82%, and 75% in the three arms. Grade ≥3 AEs occurred in 44% (long intense), 27% (intermediate), and 15% (short intense) of patients. The most frequent grade 3–4 AEs were hypertension and hyperglycemia. The subcutaneous formulation of daratumumab is also being explored in a randomized phase III trial against active monitoring in this setting (NCT03301220). Another anti-CD38 mAb, isatuximab, is under evaluation (NCT02960555).

More intense regimens using three- or four-drug combinations ±ASCT were used in the high-risk SMM setting, aiming at the eradication of MM.

In a small cohort of 12 high-risk SMM patients, Korde and colleagues demonstrated that carfilzomib–lenalidomide–dexamethasone (KRd) induced deep responses (≥CR 100%) and MRD negativity (92% by MFC); after a median follow-up of 15.9 months, none of the patients progressed to MM [53]. Interestingly, the same regimen administered in the NDMM setting produced a lower rate of deep responses (≥CR 56%), suggesting that high-risk SMM patients can be more sensitive to treatment [53]. 

In the single-arm, phase II GEM-CESAR trial, patients received KRd as induction, ASCT, KRd as consolidation and maintenance therapy with Rd. After a median follow-up of 32 months (8–128), the 30-month PFS was 93% and each phase of therapy was associated with increasing rates of MRD negativity evaluated by NGF (sensitivity 3 × 10^−6^; 31% after induction, 56% after ASCT, 63% after consolidation). During induction, the most common G≥3 AEs were infections (18%), skin rash (9%), neutropenia (6%), and thrombocytopenia (11%). Cardiac AEs were rare: 1 grade 1 atrial fibrillation, 1 cardiac failure secondary to respiratory infection, and 3 cases of hypertension during consolidation [54].

Another ongoing randomized phase II study (HO147SMM) is comparing KRd to Rd, but no data are available yet. 

The addition of the anti-CD38 mAb daratumumab to KRd induction and consolidation is being evaluated in the ASCENT study (NCT03289299), which is now recruiting patients. A randomized comparison of daratumumab–Rd vs. Rd in the context of high-risk SMM is also ongoing (NCT03937635).

## 4. Treatment of Symptomatic NDMM 

The first efforts aiming at a curative approach in NDMM were done by the University of Arkansas group in the 1990s, developing a program called Total Therapy (TT) using a series of non-cross-resistant induction regimens, 2 cycles of high-dose chemotherapy, followed by ASCT and maintenance treatment [63]. Toxicity concerns and the unavailability of novel agents hindered the success of this approach, although the long-term follow-up of treated patients (median 21 years) showed a plateau in the survival curves with an estimated cure rate of 9% based on PFS data and of 18% based on the duration of CR [64].

Currently, general treatment approaches in NDMM patients are tailored upon their eligibility for high-dose therapy (HDT) and ASCT [65].

### 4.1. ASCT-Eligible Patients

The current therapeutic approach includes sequential induction therapy and ASCT ± consolidation, followed by maintenance. Induction is typically administered for 4–6 cycles prior to ASCT [66]. The introduction of the PI bortezomib increased the response rate compared to classical chemotherapy [67], and is now a backbone of treatment. The addition of a third drug to the bortezomib–dexamethasone (Vd) combination (i.e., thalidomide [VTd], cyclophosphamide [VCd], lenalidomide [VRd], doxorubicin [PAD]) increased the depth of response [68]. In a head-to-head comparison, VTd was superior to VCd as induction prior to HDT–ASCT in terms of ORR (92% vs. 83%) and ≥VGPR rate (66% vs. 56%) [69], demonstrating that even with first-generation novel agents, the combination of a PI and an IMiD was beneficial.

In phase III trials, VRd induction was tested in the PETHEMA and Intergroupe Francophone du Myélome (IFM) 2009 studies (Table 4). 

No randomized trial directly compared VRd vs. VTd induction, although a recent integrated analysis of French and Spanish trials was performed (VRd: PETHEMA, GEM 2012, and IFM 2009; VTd: GEM2005 and IFM 2013-04) [79]. In the Spanish studies, after 6 cycles of induction, the ≥VGPR rate was 66.3% vs. 51.2% (*p* = 0.003) in VRd vs. VTd groups. In the French studies, after 4 cycles of induction, the ≥VGPR rate was similar between VRd vs. VTd groups (57.1% vs. 56.5%). The safety profile of VRd was better than that of VTd in both Spanish and French studies, with a lower rate of polyneuropathy (PNP). 

High-dose melphalan (200 mg/m^2^, MEL200) followed by ASCT is currently a standard approach in transplant-eligible patients, due to the longer PFS showed in randomized clinical studies comparing ASCT vs. novel agent-based therapy [50,74,80,81], but the role of double vs. single ASCT is still an open issue. The EMN02/HO95 phase III trial showed a benefit in the double ASCT arm in terms of PFS (3-year PFS 73% vs. 64% in double vs. single ASCT); this effect was particularly evident in the high cytogenetic risk group, where an OS benefit was also noticed [82]. Similarly, in a meta-analysis including three phase III trials, after a median follow-up of 10 years, double ASCT was significantly better than single ASCT in terms of PFS and OS. Consistent with the EMN02/HO95 data, the benefit was particularly evident in the high-risk group [82], suggesting that, in this patient population, a double ASCT is advisable. Nevertheless, the STAMINA trial did not show any difference in PFS or OS of patients receiving double vs. single ASCT. It is always difficult to perform comparisons between different trials, but the better and prolonged induction (VRD) used in the majority of the patients enrolled in the STAMINA study (whereas 3-4 cycles of VCD were used in the EMN02 study) and the lower compliance to the second ASCT procedure reported in the same study can partially explain the different results [83].

Many trials explored consolidation regimens with the rationale to deepen patient response. In the most recently published PETHEMA study, VRd induction, ASCT, and VRd consolidation produced a ≥CR rate of 58% (46% sCR, 12% CR) [75]. These data are consistent with the IFM phase II and phase III studies using VRd consolidation. In the IFM2009 study, VRd consolidation after VRd induction and ASCT showed a similar trend, with the ≥CR rate increasing from 27% during the induction phase, to 47% after ASCT to 50% after consolidation (sCR 40%, CR 10%) (Table 4) [70]. Response deepened over time, as well as MRD negativity. In the PETHEMA study, MRD by NGF with a cut-off sensitivity of 3 × 10^−6^ progressively increased from 34.5% post-induction to 53.4 % post-ASCT, to 58% after consolidation [75]. The phase III STAMINA and EMN02/HO95 trials were designed to evaluate the role of consolidation vs. no consolidation in a randomized fashion. In the STAMINA trial, the 38-month probability for PFS was respectively 58% with single ASCT + VRd consolidation, 58% with tandem ASCT and no consolidation, and 53% with single ASCT and no consolidation, with no statistical differences [83]. In the EMN02/HO95 study, VRd consolidation after ASCT/bortezomib–melphalan–prednisone (VMP) showed a PFS advantage, with a 5-year PFS of 48% in the VRd consolidation arm and 41% in the no consolidation arm [83,84]. 

In transplant-eligible patients, maintenance therapy is the standard approach after ASCT ± consolidation. A meta-analysis of three phase III trials randomizing patients to lenalidomide vs. observation/placebo showed a significant benefit in the lenalidomide arm in terms of PFS (median, 53 months vs. 24 months, HR 0.48; *p* = 0.001) and OS (not reached (NR) vs. 86 months, HR 0.75; *p* = 0.001) [85]. More recently, the Myeloma XI study confirmed the advantage of lenalidomide maintenance vs. observation after ASCT (median PFS 57 vs. 30 months, HR 0.48, *p* < 0.0001; 3-year OS 87.5% vs. 80.2%, HR 0.69, *p* = 0.01) [86]. Maintenance with lenalidomide can also further deepen the response, with 27–30% of MRD-positive patients becoming MRD-negative during treatment [87]. Besides its efficacy, the tolerability of continuous lenalidomide maintenance is an important issue. In the meta-analysis, about 30% of subjects receiving lenalidomide experienced a treatment-related AE that led to discontinuation. Moreover, a higher incidence of SPMs in the lenalidomide arm was reported, although it was outweighed by the advantage of a better disease control [85]. Although the optimal duration is currently considered to be until progressive disease, the median actual duration is generally around 2–3 years [85], with retrospective data showing a benefit in patients continuing the drug for at least 2 years [88,89]. However, there are currently no randomized prospective data showing evidence that lenalidomide until progressive disease is better than its administration for a prolonged but fixed duration [74]. 

Maintenance with lenalidomide alone showed conflicting results in high-risk patients [85,86], and the addition of PIs was suggested to be beneficial [90]. In a phase III trial [91], long-term treatment with bortezomib showed to abrogate the negative effect of deletion 17p [92,93,94]. Moreover, in a randomized study, the administration of the second-generation PI ixazomib as post-ASCT maintenance improved PFS compared to placebo and showed a similar effectiveness for both standard- and high-risk patients [95].

The high rate of deep responses (CR and MRD negativity) obtained after this sequential first-line treatment could further be improved by incorporating the second-generation irreversible PI carfilzomib or adding a fourth drug class, such as the anti-CD38 mAbs. 

The incorporation of carfilzomib into first-line treatment was tested in several trials (Table 4) [53,96,97,98]. In the randomized phase II FORTE trial, carfilzomib was combined either with lenalidomide (KRd) or cyclophosphamide (KCd) with or without ASCT (arm A KCd–ASCT–KCd; arm B KRd–ASCT–KRd; arm C KRd-12 cycles), followed by maintenance with KR or R. After a median follow-up of 26 months, the post-consolidation response rates and MRD negativity were significantly higher in the two KRd arms (B and C) than in the KCd arm (A): ≥VGPR rate was 74% (arm A), 87% (arm B), and 87% (arm C), and the ≥CR rate 38% (arm A), 50% (arm B), and 52% (arm C). The MRD negativity rate by MFC 10^−5^ after consolidation was respectively 41% (arm A), 58% (arm B), and 54% (arm C) [99]. The main non-hematologic grade ≥3 AEs were hypertension (8% KRd-12 vs. 3% KRd–ASCT and KCd–ASCT), cardiac AEs (2% KRd-12 vs. 3% KRd–ASCT vs. 3% KCd–ASCT), infections (13% KRd-12 vs. 10% KRd–ASCT vs. 9% KCd–ASCT), and hepatic AEs (10% KRd-12 vs. 8% KRd–ASCT vs. 1% KCd–ASCT) [99].

Despite similar MRD negativity rates, a lower number of early relapsing patients was noted in the KRd–ASCT arm than in the KRd-12 arm. This was observed in intermediate + high-risk patients, but not in standard-risk patients, suggesting that, despite the use of second-generation PIs upfront, ASCT could still play a role in this patient population [9]. 

The addition of an anti-CD38 antibody to triplet regimens has been explored in several trials as well. In the phase III trial CASSIOPEIA, daratumumab–VTd (Dara–VTd) induction-ASCT–Dara–VTd was superior to VTd-ASCT-VTd in terms of response rate after consolidation, with ≥VGPR rate of 83% vs. 78%, CR rate of 10% vs. 6%, and sCR rate of 28.9% vs. 20.3%. MRD negativity (10^−5^) after consolidation was reached in 64% vs. 44% of patients in the Dara–VTd vs. VTd arms; PFS was significantly improved in the Dara–VTd group, as compared with the control group (HR 0.47, 95% confidence interval (CI) 0.33–0.67, *p* < 0.0001) [77].

The phase II GRIFFIN study compared Dara–VRd to VRd alone (Table 4). Dara–VRd improved the sCR rate by end of consolidation (42.4% vs. 32.0%). Overall, post-consolidation response was better in the Dara–VRd arm (≥VGPR 91%, ≥CR 52%, of which 59% MRD-negative) compared to the VRd arm (≥VGPR 73%, ≥CR 42%, of which 24% MRD-negative); MRD negativity was achieved in 44% of patients in the Dara–VRd arm after consolidation (10^−5^ threshold by NGS) [76].

A phase Ib study evaluated the addition of daratumumab to carfilzomib-based induction (Dara–KRd). Serious AEs occurred in 46% of patients. The most common grade 3–4 AEs were lymphopenia (50%) and neutropenia (23%); 1 cardiac grade 3 AE was observed (congestive heart failure). In 22 treated patients, ORR was 100% (CR 5%, ≥VGPR 86) [11].

Similarly, the addition of isatuximab to KRd is being investigated in the phase II GMMG-CONCEPT study. In the safety run-in phase (10 patients), the overall safety profile was consistent with those previously reported with KRd and isatuximab. Non-hematologic grade ≥3 AEs were treatment-unrelated cerebral vascular disorder (2 patients), self-limiting ventricular tachycardia (1), and diarrhea (1). Three patients experienced a grade 2 infusion-related reaction (IRR) during the first infusion of isatuximab [100].

Quadruplet regimens not including mAbs may allow to achieve deep responses in the majority of patients, preserving the opportunity to use mAbs after induction ± ASCT in patients not achieving MRD negativity. Bortezomib–lenalidomide–cyclophosphamide–dexamethasone (VRCd) produced a ≥VGPR rate of 33% after 4 induction cycles [71], while carfilzomib–lenalidomide–cyclophosphamide–dexamethasone (KRCd) produced a ≥VGPR rate of 82% (MRD negativity 55% at 10^−4^–10^−5^ sensitivity by flow cytometry) after a median of 4 induction cycles (range 1–12) in transplant-eligible patients [72].

In a small group of patients, the addition of the second-generation oral PI ixazomib to Rd (Ixa–Rd) during induction followed by ASCT or by ixazomib maintenance induced a good response, with 63% of patients achieving ≥VGPR and 12% MRD negativity. However, responses were not as deep as those reached in patients treated with upfront daratumumab or carfilzomib, making Ixa–Rd less appealing from a curative perspective [78].

### 4.2. ASCT-Ineligible Patients

ASCT-ineligible patients are a heterogeneous population. Scores predicting mortality and the risk of treatment toxicity in elderly patients have been assessed. Evidence from clinical trials [101] suggested that frailty-adapted therapies should be applied and that mainly fit patients can benefit from strategies aiming at the deepest possible response, due to higher toxicities with similar therapies in intermediate–fit/frail patients that in the end hamper the effectiveness of treatment itself [102,103].

The standard first-line treatment schemes for elderly patients are VMP, Rd, and VRd. In the phase III VISTA trial, VMP was superior to melphalan-prednisone (MP) in terms of CR rate, PFS, and OS (median 56 months vs. 43 months) [104,105].

Continuous Rd significantly increased PFS and OS compared to MPT and also prolonged PFS (but not OS) compared to Rd18 (median PFS 26 months for Rd vs. 21 months for Rd18 and 21.9 months for MPT; 4-year estimated OS 59% for Rd vs. 56% for Rd18 and 51% for MPT). Rd was also generally better tolerated than MPT [106]. In a phase III clinical trial specifically designed for intermediate–fit patients, according to the IMWG frailty score, continuous Rd was compared to Rd induction for 9 cycles followed by R maintenance alone at lower doses: PFS was superimposable, with a better tolerability with Rd–R [107].

VRd was also prospectively compared to Rd in the SWOGS0777 trial (Table 5), which, however, was not restricted to elderly patients (median age 63 years) [108]. The addition of bortezomib to Rd resulted in significantly improved PFS (43 months vs. 30 months in the Rd group; *p* = 0.0018) and OS (75 months vs. 64 months in the Rd group; *p* = 0.025)**.** Regarding safety, the VRd combination showed higher rates of grade ≥3 AEs (82 vs. 75%), neurological toxicities (33% vs. 11%), and discontinuation (23% vs. 10%). The high neurological toxicity could be due to the two-weekly intravenous infusion of bortezomib used in this trial. In a small phase II study [109], a modified VRd, including lower lenalidomide doses (15 mg) and weekly subcutaneous bortezomib (“VRd lite”), produced a median PFS of 35.1 months and fewer toxic effects. 

Studies exploring the upfront use of anti-CD38 mAbs in transplant-ineligible patients showed deep responses also in this setting. In the ALCYONE trial, the quadruplet daratumumab–VMP (Dara–VMP) was compared to VMP showing a clear advantage in PFS (HR 0.50, 95% CI 0.38–0.65, *p* < 0.001) [27]. At least CR rates were 42 vs. 24% and MRD negativity rates by NGS were 22.3% vs. 6.2%, respectively. Safety issues mostly consisted of IRRs (overall 27%, grade ≥3 5%) and a high incidence of infections (grade ≥3 pneumonia 11% vs. 4% in the Dara–VMP vs. VMP arms). 

Similarly, in the phase III randomized MAIA study, Dara–Rd significantly prolonged PFS as compared to Rd (HR 0.56, 95% CI 0.43–0.73, *p* < 0.001), with ≥CR rates of 47% vs. 24% and MRD negativity in 24% vs. 7% patients, respectively. The safety profile was similar in the two arms, but the daratumumab group experienced a higher incidence of neutropenia and infections (including pneumonia) than the Rd group. As in the ALCYONE study, IRRs were reported in the daratumumab arm (overall 40%, mostly of grades 1–2 with an incidence of grade ≥3 IRRs of 2.7%) [110]. 

An ongoing phase I study is investigating isatuximab, in combination with VRd (Isa–VRd): the first report on 22 patients showed good tolerability, with 46% of grade ≥3 AEs, mostly hematologic. Besides, response rates are promising, with MRD negativity rates (10^−6^) by NGS of 33% and by NGF 18% [111].

The good results from the upfront use of second-generation PIs in the transplant-eligible setting encouraged its exploration in several clinical trials for the treatment of elderly patients. Carfilzomib associated with melphalan and prednisone (KMP) showed promising results in a phase I/II study, with 90% ORR and 58% ≥VGPR rates and about 8% of grade ≥3 cardiovascular AEs [112]. However, in the phase III CLARION study, KMP failed to outperform VMP in terms of PFS, OS and MRD negativity rates [113]. The safety profile was different between the two arms, with KMP inducing more acute renal failure (any grade 13.9% vs. 6.2%), more cardiac failure (any grade 10.8% vs. 4.3%), and less peripheral neuropathy (grade ≥2 2.5% vs. 35.1%) than VMP. 

The association of carfilzomib with cyclophosphamide dexamethasone (KCd) was evaluated in two phase I/II studies, the first adopting the once-weekly carfilzomib schedule and the second the twice-weekly schedule [114,115]. Both trials demonstrated a high efficacy profile (median PFS 35.7 and 35.5 months, respectively; 3-year OS: 72% and 75%) with acceptable toxicity. Overall toxicities mainly occurred during the induction phase and the incidence of non-hematologic AEs was similar to that observed with the KMP combination. KCd showed a lower myelotoxicity than KMP and VMP. Of note, few AEs emerged during maintenance. The once- and twice-weekly schedules were compared in a meta-analysis, with no significant differences in terms of efficacy and toxicities, and with a benefit also observed in high-risk patients [116].

## 5. Future Perspectives

**Figure 1:**
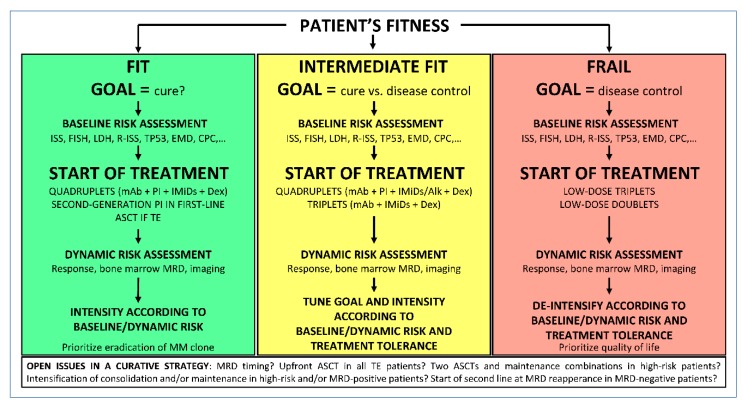
Proposed algorithm to set treatment goal in NDMM patients. ISS, international staging system; FISH, fluorescence in-situ hybridization; LDH, lactate dehydrogenase; R-ISS, revised ISS; EMD, extramedullary disease; CPC, circulating plasma cells; mAb, monoclonal antibody; PI, proteasome inhibitor; IMiDs, immunomodulatory drugs; ASCT, autologous stem cell transplantation; TE, transplant eligible; MRD, minimal residual disease.

Patient fitness is one of the first factors to consider when planning the treatment strategy. Despite the manageable profile of some effective combinations, frail patients can unlikely tolerate full-dose combinations that may induce high MRD negativity. In these patients, disease control rather than cure may be the more realistically achievable goal. Nevertheless, disease control lasting for a few years, even without achieving CR or MRD negativity, could allow very elderly patients to have the same survival of age-matched healthy subjects, considering their actual life expectancy. On the other hand, in fit patients, the outcome-limiting factor is usually disease progression, and a curative approach aiming at sustained MRD negativity could be pursued. This approach should incorporate baseline risk evaluation and dynamic risk evaluation (MRD achievement and duration) during treatment (Figure 1).

Baseline risk factors such as International Staging System (ISS), cytogenetics, lactate dehydrogenase (LDH) levels [117], extramedullary disease [118], circulating plasma cells [119], TP53 mutations [94], and many others can help define our therapeutic strategy. For instance, the use of double ASCT and long-term treatment with a PI plus IMiDs maintenance could be beneficial in the presence of high-risk cytogenetics [92]. The dynamic evaluation of patient risk after the start of treatment can also help tune treatment intensity. Many MRD-driven therapeutic choices are under investigation in clinical trials. One possibility is to evaluate treatment escalation in patients who do not achieve MRD negativity at a pre-specified time point. In particular, this could be the approach for high-risk aggressive MM, mirroring a strategy such as the one used for acute leukemia, where achieving MRD is the goal to achieve cure. Another possibility is to evaluate treatment withholding in patients with sustained MRD negativity. This could be the option with standard-risk disease, where the disease behavior is more similar to that of chronic leukemias. In MRD-negative patients, if a reappearance of MRD is detected, restarting prior therapy if previously interrupted, or starting a different second-line therapy before the development of an overt relapse, can also be explored. Of note, the deferral of treatment is currently recommended, even at the reappearance of a monoclonal component (biochemical relapse), if the increase of the monoclonal component is slow [120]. Treating the reappearance of MRD might be a further step for prolonged disease control but its usefulness should be demonstrated in well-designed trials.

Before using MRD in the treatment of MM, several questions need to be answered. The first question is in which patients we should test MRD (in CR or sCR patients only, or in VGPR patients). The rationale to test MRD in VGPR patients is that, due to the long half-life of serum immunoglobulin (~1 month), the complete clearance of monoclonal component could take months until all the cells producing it have been eradicated, especially in IgG cases [121]. In these cases, VGPR patients who are MRD-negative in the bone marrow achieve CR in the months following MRD testing. However, MM is a spatially heterogeneous disease and residual plasma cells in extramedullary sites can produce the monoclonal component in VGPR cases in the presence of MRD negativity in the bone marrow. If this is the case, MRD should probably be measured at sCR and the confirmation of bone marrow MRD negativity with imaging techniques should be performed. In the context of a MRD-driven therapy, it is also tricky to evaluate (a) the impact and the likelihood of “false negative” or “false positive” MRD results; (b) the right time point; (c) the reasonably achievable cut-off at a specific time point. For instance, in the transplant-eligible setting, the post-induction time point could be used to investigate different durations and/or intensifications of induction regimens, and to understand whether or not intensification with transplant is necessary. It is possible that, after different treatments, different cut-offs can be achievable. As an example, a 10^−5^ negativity can be the reasonable goal after induction, but with prolonged intensification (transplant or further consolidation), a deepest MRD negativity should likely be the goal. This means that different MRD cut-offs at different time points should be considered in planning MRD-driven treatment strategies. Moreover, the question is if treatment decision can rely on a single MRD evaluation, or if, as for all the other response categories, MRD needs to be confirmed. This also affects the choice of the best time point (for instance, can we reasonably use a post-induction time point to make decisions on treatment intensification if we consider an induction with 4 cycles only?). Secondly, we should also consider the importance of MRD duration, particularly in the context of continuous therapy.

In the transplant-eligible setting, a further issue is the role of checking and the feasibility of pursuing MRD negativity in the peripheral blood stem-cell collection. Autografts contaminated with MM cells (MRD-positive autografts at a sensitivity of 10^−7^ by NGS) predicted a worse PFS than MRD-negative autografts [122]. However, this effect was mitigated in patients receiving further treatment after ASCT. Indeed, it should be noted that ASCT, consolidation and maintenance, especially with drugs not used during induction, still have the potential to eradicate MRD in a substantial number of patients who are MRD-positive at post-induction time point [7,8,43,70,123]. 

While the achievement of MRD negativity is clearly predictive of good outcomes, some NDMM patients are characterized by an MGUS-like plasma cell compartment [124]. In these patients, long-term disease control can be accomplished without achieving deep responses, probably due to an immune control of the residual disease. Further research is needed to reliably identify this patient population. 

Moreover, the restoration of a physiological immune system at the time of MRD assessment could also play a role in predicting the patients that will possibly not relapse [28].

## 6. Conclusions

In conclusion, we are living exciting times in the field of MM, with many new regimens and strategies in the pipeline and an increasing knowledge of the complexity of the disease. Even if we currently do not have any evidence that we are able to cure MM in the great majority of treated patients, the longer follow-ups of the recent studies will determine the percentage of subjects able to actually maintain a disease-free status for a very long time. New well-designed MRD-driven trials will help us determine if it will be worth aiming at the cure of the disease and what will be the best therapeutic approach to achieve it.

## Figures and Tables

**Table 1:** Comparison of next-generation sequencing (NGS) and next-generation flow (NGF) for the detection of minimal residual disease (MRD) in multiple myeloma.

Characteristics	NGS	NGF
**Applicability**	≥90%	~100%
**Baseline sample**	Required for molecular marker identification	Not required
**Number of cells**	1–2 million cells/20 µg DNA	10 million cells/tube
**Standardization**	Commercial companies (e.g., Adaptive Biotechnologies)	Euro Flow consortium
**Sample processing**	Fresh and/or stored samples	Fresh sample required; processing within ≤48 h
**Sample quality control**	Not feasible	Possible to check by global bone marrow cell analysis
**Quantitative**	Yes	Yes
**Sensitivity**	1 in 10^−5^–10^−6^	1 in 10^−5^–10^−6^
**Clonal evolution**	Evaluable	Not evaluable
**Time required**	1 week	3–4 h
**Support required**	Bioinformatics support	Expert flow cytometrist; Automated software

MRD, minimal residual disease; NGS, next-generation sequencing; NGF, next-generation flow.

**Table 2:** Smoldering multiple myeloma: risk stratification systems.

Stratification System	*N*	Low Risk		Intermediate Risk				High Risk	
		Criteria	TTP (Median)	Criteria		TTP (Median)		Criteria	TTP (Median)
Mayo Clinic [47]	**273**	**- M-protein <3 g/dL** **plus** **- BMPCs <10%**	19 years	- M-protein <3 g/dL plus - BMPCs ≥10%		8 years		- M-protein ≥3 g/dL plus - BMPCs ≥10%	2 years
PETHEMA [48]	89	- Aberrant PCs by MFC <95% - Immunoparesis	NR	- Aberrant PCs by MFC ≥95% OR - Immunoparesis		6 years		- Aberrant PCs by MFC ≥95% AND - Immunoparesis	1.9 years
Mayo 2/20/20 [45]	421	None of the risk factors: - M-protein >2g/dL - BMPCs >20% - FLCr >20	9.1 years	One risk factor: - M-protein >2g/dL - BMPCs >20% - FLCr >20		5.6 years		≥2 risk factors: - M-protein >2g/dL - BMPCs >20% - FLCr >20	2.4 years
		**Low Risk**		**Intermediate Low**		**Intermediate High**		**High Risk**	
	***N***	**Criteria**	**2-year progression risk**	**Criteria**	**2-year progression risk**	**Criteria**	**2-year progression risk**	**Criteria**	**2-year progression risk**
2/20/20 + CA by FISH * [49]	2004	None of the risk factors: - M-protein >2g/dL - FLCr >20 - BMPCs >20% Presence of any of the CA *	8%	1 risk factor: - M-protein >2g/dL - FLCr >20 - BMPCs >20% Presence of any of the CA *	21%	2 risk factors: - M-protein >2g/dL - FLCr >20 - BMPCs >20% Presence of any of the CA *	37%	≥3 risk factors: - M-protein >2g/dL - FLCr >20 - BMPCs >20% Presence of any of the CA *	59%

* High-risk chromosomal abnormalities: t(4,14), t(14,16), 1q gain, or del13. N, number; M-protein, myeloma protein; BMPCs, bone marrow plasma cells; CA, chromosomal abnormalities; FLCr, free light chain ratio; MFC, multiparameter flow cytometry; NR, not reached; PCs, plasma cells; TTP, time to progression; FISH, fluorescence in situ hybridization.

**Table 3:** Smoldering multiple myeloma: Selected clinical trials.

Protocol	Phase	Treatment		Pts	Response	TTP/PFS/OS	Toxicity (≥G3)
NCT00480363 QuiRedex [52]	Phase III	Rd vs. observation	Induction: 28-day cycle (cycles 1–9) lenalidomide 25 mg p.o. days (D) 1–21 + dex 20 mg p.o. D1–4, 12–15 Maintenance: (cycles 1–24) 28-day cycle Lenalidomide 10 mg D1–21 vs. Observation	119	FU 73 months Best response 26% CR after maintenance	Median TTP NR (HR 0.24) 3-year PFS 77% 3-, 5-year OS 94%, 88% (HR 0.43) vs. median TTP 23 months (*p* < 0.001) 3-year PFS 30% (*p* < 0.001) 3-, 5-year OS 80%, 71%, (*p* = 0.03)	Neutropenia 5% Thrombocytopenia 2% Anemia 2% Infection 6% Asthenia 6% Skin rash 3% vs. none
NCT01572480 120107 [53]	Phase II	KRd induction Rd maintenance	Induction: 28-day cycle (cycles 1–8) carfilzomib 20/36 mg/m^2^ iv D1, 2, 8, 9, 15, 16 + lenalidomide 25 mg p.o. D 1–21 + dex 20 mg (cycles 1–4) and 10 (cycles 5–8) p.o. or iv D1, 2, 8, 9, 15, 16 Maintenance (cycles 1–24): 28-day cycle lenalidomide 25 mg D1–21	12	CR 100%. MRD neg (MFC 10^−5^ ) 92%	No progression to MM 3-, 4-year PFS 94%, 70.6% 3-, 4- year OS: 100%	Hematologic: Lymphopenia 39% Neutropenia 28% Anemia 22% Thrombocytopenia 11% Non-hematologic: Diarrhea 17% Lung infection 17% Hypophosphatemia 11% 1 case of CHF
NCT02415413 GEM-CESAR [54]	Phase II	KRd induction ASCT KRd consolidation Rd maintenance	Induction: 28-day cycle (cycles 1–6): carfilzomib 20/36 mg/m^2^ iv D1, 2, 8, 9, 15, 16 + lenalidomide 25 mg p.o. D1–21 + dex 40 mg D1, 8, 15, 22 ASCT melphalan 200 mg/m^2^ Consolidation: KRd 2 cycles Maintenance: 28-day cycle lenalidomide 10 mg days 1–21 + 20 mg days 1, 8, 15, 22 for 2 years	90	ORR: 100%. ≥CR: 70% MRD (NGF) 57%	30 months PFS 98%	Induction (G3-4): Neutropenia 6% Thrombocytopenia 11% Infections 18% Consolidation: Ongoing
NCT03673826 HO147SMM	Phase II	KRd vs. Rd Rd maintenance	Induction: (cycles 1–9) carfilzomib 20/36 mg/m^2^ iv D1, 2, 8, 9, 15, 16, lenalidomide 25 mg p.o. D1–21, dexamethasone 20 mg p.o. (cycles 1–4) and 10 mg p.o. (cycles 5–9) D1, 8, 15, 22 vs. (cycles 1–9) lenalidomide 25 mg p.o D1–21, dexamethasone 20 mg p.o. (cycles 1–4) and 10 mg p.o. (cycles 5–9) D1, 8, 15, 22 Maintenance: (cycles 1–24) lenalidomide 10 mg p.o. days 1–21 of a 28-day cycle	120	NA	NA	NA
NCT03289299 ASCENT	Phase II	Dara KRd + Rd maintenance	Induction: 28-day cycle (cycles 1–6) carfilzomib 20/36 mg/m^2^ iv D1, 2, 8, 9, 15, 16 + lenalidomide 25 mg p.o. D1–21 + dex 40 mg D1, 8, 15, 22, daratumumab 16 mg/kg iv D1, 8, 15, and 22 of cycles 1–2; D1 and 15 of cycles 3–6; Consolidation: 6 cycles, KR as in induction + dex 20 mg p.o. D1, 8, 15, 22 Daratumumab 16 mg/kg iv D1 of cycles 7–12 vs. ASCT Maintenance: 12 cycles, lenalidomide 10 mg p.o. D1–21 + daratumumab; 16 mg/kg iv D1 of odd cycles for cycles 13–24	83	NA	NA	NA
NCT02697383 15-294 [55]	Phase I	Ixazomib dexamethasone	28-day cycle (cycles 1–12) ixazomib 4 mg on D1, 8, and 15, and dexamethasone on days 1, 8, 15, and 22 (40 mg/week the first 4 cycles, thereafter 20 mg/week)	14	ORR 64%	64% ORR (8 PR, and 1 VGPR) no patient progressed to MM	Lung infection (14%)
NCT01441973 CA204-011 [56]	Phase II	Elotuzumab	(cycle 1) elotuzumab 20 mg/kg iv D1, 8 then [cycle 2—progressive disease] Elotuzumab monthly q4 week vs. (cycle 1) elotuzumab 10 mg/kg iv D1, 8, 15, 22 (cycle 2—progressive disease) elotuzumab monthly q2 week	31	Both groups ORR 10%	Both groups 2-year PFS 69%	Upper respiratory tract infection 7% vs. Fatigue 6% Diarrhea 6% Insomnia 6%
NCT02279394 14-338 [57]	Phase II	Elotuzumab Rd + stem-cell mobilization + maintenance	Induction: 28-day cycle (cycles 1–2) elotuzumab 10 mg/kg iv D1, 8, 15, 22 + lenalidomide 25 mg p.o. D1–21 + dex 40 mg p.o. D1, 8, 15, 22 (cycles 3–8): elotuzumab 10 mg/kg iv D1, 15 + lenalidomide as in cycles 1–2 + dex 40 mg p.o. D1, 8, 15 Maintenance: 28-day cycle (cycles 9–4) elotuzumab 10 mg/kg iv D 1 + lenalidomide as in cycles 1–2	50	≥VGPR 43%	NA	Hypophosphatemia 34% Neutropenia 26% Lymphopenia 22%
NCT02916771 16-313 [58]	Phase II	Ixazomib-Rd	Induction: 28-day cycle (cycles 1–9): ixazomib 4 mg p.o. D1, 8, 15 + lenalidomide 25 mg p.o. D1–21 + dex 40 mg p.o. D1, 8, 15, 22 Maintenance: 28-day cycle (cycles 10-24): ixazomib 4 mg p.o. D1, 8, 15 + lenalidomide 25 mg p.o. D1–21	26	≥VGPR 53.8%	NA	Hypophosphatemia 13% Lymphopenia 13% Neutropenia 8.7% (G4 Neutropenia in 1 pt)
NCT01484275 CR100755 [59]	Phase II	Siltuxumab vs. placebo	(cycle 1—until progressive disease): siltuximab 15 mg/kg iv every 4 weeks vs. observation	87	NA	1-year PFS: 84% Median PFS: NR vs. 1-year PFS: 74.4% Median PFS: 23.8 months	Infections (5 patients in siltuximab group and 6 patients in placebo group) Urinary disorders (one patient in the siltuximab group and three patients in the placebo group)
NCT02960555 2015-0148	Phase II	Isatuximab	(cycles 1–30): 28-day cycle isatuximab iv D1, 8, 15, and 22 of cycle 1, on D1 and 15 of cycles 2–6, and on D1 of subsequent courses	61	NA	NA	NA
NCT02316106, CENTAURUS [60]	Phase II	Daratumumab iv (3 arms, 41 patients each)	Daratumumab 16 mg/kg iv in 8-week cycles Long intensity:(cycle 1) every 1 week; (cycle 2–3) every other week; (cycles 4–7) every 4 weeks; (cycles 8–20) every 8 weeks Intermediate intensity: (cycle 1) every 1 week and (cycles 2–20) every 8 weeks Short intensity: (cycle 1) every week for 8 infusions	123	≥CR rate 7% in combined Long and Int arms	Long intensity 24-month PFS 90% vs. intermediate intensity 24-month PFS 82% vs. short intensity 24-month PFS 75%	Long intensity serious adverse events, 32% ≥G3 AE 44% - Hypertension 7% - Hyperglycemia 2% vs. intermediate intensity serious adverse events 15% ≥G3 AE 27% - Hypertension 5% - Hyperglycemia 5% vs. short intensity serious adverse events, 10% ≥G3 AE 15% - Hypertension 3% - Hyperglycemia 0%
NCT03301220 AQUILA [61]	Phase III	Daratumumab sc for 3 years vs. observation	Daratumumab sc injection (daratumumab 1800 mg + rHuPH20 [2000 U/mL]) once weekly for cycles 1 and 2 (D1, 8, 15, and 22 of each week), every 2 weeks for cycles 3–6 (D1 and 15), and thereafter every 4 weeks (D1) until 39 cycles or up to 36 months or PD vs. observation	390	NA	NA	NA
NCT01169337 E3A06 [62]	Phase II–III	R vs. observation (median FU 35 months)	Lenalidomide 25 mg p.o. D1-21 in 28 days cycle until PD vs. observation	180	Overall response 47.7% phase II 48.9% phase III	PFS 1 year (98% vs. 89%), PFS 2 years (93% vs. 76%) PFS 3 years (91% vs. 66%)	G3/4 non-hematologic AEs 28%: - Infections 10% - Fatigue 6.8% - Hypertension 9% G4 hematologic AEs 4.5%, primarily neutropenia (*n* = 4), cumulative incidence of invasive SPMs 11.4% (Len) and 3.5% (observation).

D, day; Pts, patients; R, Len, lenalidomide; d, dex, dexamethasone; FU, follow-up; TTP, time to progression; PFS, progression-free survival; OS, overall survival; G, grade; P, *p*-value; MM, multiple myeloma; K, carfilzomib; ORR, overall response rate; Dara, daratumumab; ASCT, autologous stem-cell transplantation; NA, not available; NR, not reached; iv, intravenous; D, day; AE, adverse event; sc, subcutaneous; p.o., orally; HR, hazard ratio; CR, complete response; PR, partial response; VGPR, very good PR; PD, progressive disease; CHF, congestive heart failure; SPMs, second primary malignancies; MRD, minimal residual disease; neg, negative.

**Table 4:** Newly diagnosed multiple myeloma: selected clinical trials enrolling transplant-eligible patients.

Protocol	Phase	Treatment		Subjects	Response	TTP/PFS/OS	Toxicity (≥G3)
NCT01206205 IFM2008 [70]	Phase II	VRd induction Stem-cell mobilization ASCT VRd consolidation R maintenance	Induction 21-day cycles (cycles 1–3) Bortezomib iv 1.3 mg/m^2^ D1, 4, 8, and 11 Lenalidomide p.o. 25 mg D1–14 Dexamethasone p.o. 40 mg D1, 8, 15 Stem-cell harvest Consolidation 21-day cycles (cycles 1–3) Bortezomib iv 1.3 mg/m^2^ D1, 4, 8, and 11 Lenalidomide p.o. 25 mg D1–14 + dex p.o. 40 mg D1, 8, 15 Maintenance Lenalidomide p.o. 10 mg per day for the first 3 months, a possible dose increase to 15 mg for 1 year	31	After induction 58% ≥VGPR 23 % ≥CR 16% MRD neg MFC (10^−4^–10^−5^) After ASCT 70% ≥VGPR 47%≥CR 54% MRD neg MFC (10^−4^–10^−5^) After consolidation 87% ≥VGPR 50 ≥CR 58% MRD neg MFC (10^−4^–10^−5^) Best response (after maintenance) 84% ≥VGPR 58% ≥ CR 68% MRD neg MFC (10^−4^–10^−5^)	Estimated 3-year PFS 77% and OS 100% (median FU 39 months)	Neutropenia 65% Thrombocytopenia 19% Anemia 3% Infections 6%
NCT00507442 EVOLUTION [71]	Phase II randomized	VRd (*n* = 42) vs. VRCd (*n* = 48) vs. VCd (*n* = 33)	Induction 21-day cycles (cycles 1–8) Bortezomib 1.3 mg/m^2^, days 1, 4, 8, 11 Dexamethasone 40 mg, D1, 8, 15 Cyclophosphamide 500 mg/m^2^, D1, 8 Lenalidomide p.o., 15 mg in VRCd and 25 mg in VRd D1–14 Maintenance 6-week cycles (cycles 1–4) Bortezomib 1.3 mg/m^2^, D1, 8, 15, 22 Stem-cell mobilization any time after 2 cycles ASCT any time after 4 cycles	140	VRd best response 61% ≥VGPR 21% CR VRCd best response 54% ≥VGPR 21% CR VCd best response 24% ≥VGPR 10% CR	1-year PFS 83% VRd 86% VRCd 93% VCd 100% VCd-mod 1-year OS estimate 92% VRCd arm 100% for the other three arms	VRd Neuropathy 17% vs. VRCd Neuropathy 13% vs. VCd Neuropathy 9%
NCT01554852 MyelomaXI [72,73]	Phase III randomized	Transplant eligible: CTd vs. CRd vs. KCRd induction CVd intensification vs. no intensification Mel200 ASCT Lenalidomide maintenance vs. observation	Transplant eligible: Induction CTd: 21-day cycle cyclophosphamide 500 mg p.o. D1, 8, 15 thalidomide 50 mg p.o. continuously D1–21 dexamethasone 40 mg p.o. D1–4, 12–15 CRd: 28-day cycle cyclophosphamide 500 mg p.o. D1, 8 lenalidomide 25 mg p.o. D1–21 dexamethasone 40 mg p.o. D1–4, 12–15 KCRd: 28-day cycle carfilzomib 36 mg/m^2^ i.v. D1, 2, 8, 9, 15, and 16 cyclophosphamide 500 mg p.o. D1, 8 lenalidomide 25 mg p.o. D1–21 dexamethasone 40 mg p.o. D1–4, 8, 9, 15, and 16 Consolidation CVd: 21-day cycles Bortezomib i.v. 1.3 mg/m^2^ D1, 4, 8, and 11 cyclophosphamide 500 mg p.o. D1, 8, and 15 Dexamethasone p.o. 20 mg D1, 2, 4, 5, 8, 9, 11, and 12 Maintenance Lenalidomide p.o. 10 mg D1–21	1056	Post-induction CTd 53% ≥VGPR 11% MRD neg (MFC 10^−4^–10^−5^) CRd 65% ≥VGPR 21% MRD neg KCRd 82% ≥VGPR 55% MRD neg	3-year PFS 50.3% CTd/CRd 64.5% KCRd	- Neutropenia 12.8% CTd 22.3% CRd 16.4% KCRd - Thrombocytopenia 1.2% CTd 2.3% CRd 8.4% KCRd
NCT01191060 IFM2009 [74]	Phase III	VRd induction arm VRd consolidation arm ASCT	Induction: 21-day cycles (cycles 1–3) Bortezomib i.v. 1.3 mg/m^2^ D1, 4, 8, and 11 Lenalidomide p.o. 25 mg D1–14 Dexamethasone p.o. 20 mg D1, 2, 4, 5, 8, 9, 11, and 12 Stem-cell mobilization with cyclophosphamide and G-CSF Consolidation (cycles 4–8) VRd reduced dex 10 mg (VRd-alone group) vs. Melphalan 200 mg/m^2^ ASCT + 2 cycles of VRd reduced dex 10 mg (transplantation group) Maintenance Lenalidomide p.o. 10 mg per day for the first 3 months, a possible dose increase to 15 mg for 1 year	700	VRd arm After induction 45% ≥VGPR After consolidation ≥69% VGPR Best response (after maintenance) 77% ≥VGPR 48% CR of which 65% MRD neg vs. VRd–ASCT After induction 47% ≥VGPR After consolidation 78% ≥VGPR Best response 88% ≥VGPR 59% CR of which 79% MRD neg	Median PFS 50 vs. 36 months OS 4years 81% vs. 82%	Neutropenia (47% vs. 92%) Gastrointestinal disorders (7% vs. 28%) Infections (9% vs. 20%)
NCT01916252 GEM2012MENOS65 [75]	Phase III	VRd 6 cycles Stem cell mobilization after 3 induction cycles Mel200-ASCT vs. BuMel-ASCT VRd 2 cycles post-transplant consolidation	Induction (cycles 1–6) Bortezomib 1.3 mg/m^2^ sc D1, 4, 8, 11 Lenalidomide 25 mg/day D1–21 Dexamethasone 40 mg on D1–4 and 9–12 at 4 weeks ASCT Melphalan 200 mg/m^2^ iv D 2 vs. Busulfan 9.6 mg/kg + Melphalan 140 mg/m^2^ Consolidation (cycles 7–8) same schedule as induction	458	After induction 29% VGPR 39% CR 28% sCR 34% MRD-neg (by NGF) After ASCT 27% VGPR 49% CR 36% sCR 53% MRD neg After consolidation 58% MRD neg	NA	Induction Neutropenia 11% Thrombocytopenia 6% Hepatic 4% Skin 3% Neuropathy 1%
NCT01029054 UMCC 2009.056 [8]	Phase I/II Phase II extended	Phase I KRd without ASCT induction and KRd maintenance Phase II KRd induction Mobilization ASCT/Mel200 KRd consolidation KRd maintenance	Phase I/II Induction (cycles 1–8) Carfilzomib iv 20/36 mg/m^2^ D1, 2, 8, 9, 15, 16 (20 mg/m^2^ given on cycle 1, D1) Lenalidomide p.o. 25 mg D1–21 Dexamethasone p.o. 40 mg/week Maintenance (cycles 9–24) Carfilzomib 36 mg/m^2^ D1, 2, 15, 16 Lenalidomide p.o. same dose of C8 D1–21 Dexamethasone p.o. 20 mg weekly Phase II Induction (cycles 1–4) Carfilzomib iv 20/36 mg/m^2^ D1, 2, 8, 9, 15, 16 (20 mg/m^2^ given on cycle 1, D1) Lenalidomide p.o. 25 mg D1–21 Dexamethasone p.o. 40 mg/week Stem-cell collection using G-CSF and plerixafor ASCT Melphalan 200 mg/m^2^ conditioning Consolidation (cycles 5–8) Carfilzomib i.v. 36 mg/m^2^ D1, 2, 8, 9, 15, 16 Lenalidomide p.o. 15 mg D1–21 (with option to escalate to 25 mg) Dexamethasone p.o. 20 mg weekly Maintenance (cycles 9–18) Carfilzomib 36 mg/m^2^ D1, 2, 15, 16 Lenalidomide p.o. same dose of cycle 8 D1–21 Dexamethasone p.o. 20 mg weekly Lenalidomide as single-agent off-study after C18.	53 (phase I/II) 76 (phase II)	Phase I/II Post-induction (C8) 55% sCR Phase II Post-consolidation (C8) 96% ≥VGPR, 73% CR 69% sCR. Post-consolidation (C8) 82% MRD neg (MFC) (*N* = 33) 66% MRD neg (NGS) (*N* = 29) Post-Maintenance (C18) 90% MRD neg (MFC) (*N* = 20) 71% MRD neg (NGS) (*N* = 16)	Phase I/II 4- year PFS 63% 4-year OS 93% Phase II 2-year PFS 97% and 2-year OS 99% (median FU 17 months)	Phase II Lymphopenia 28% Neutropenia 18% Infections 8%
NCT01402284 110221 [53]	Phase II	KRd induction R maintenance	Induction (cycles 1–8) Carfilzomib iv 20/36 mg/m^2^, D1, 2, 8, 9, 15, 16 Lenalidomide 25 mg p.o. D1–21 Dexamethasone 20 mg (cycles 1–4) and 10 (cycles 5–8) p.o. or iv D1, 2, 8, 9, 15, 16 then Maintenance (cycles 1–24) lenalidomide 25 mg D1–21	45	62% MRD neg (calculated on NGS-evaluable NDMM patients)	18-month PFS: 100% vs. 84%	Lymphopenia 34% Thrombocytopenia 11% Neutropenia 15% Anemia 12% Infection 6% Cardiac 5% Vascular 6%
NCT02203643 FORTE [9]	Phase II	KCd-ASCT-KCd (arm A), KRd-ASCT- KRd (arm B), KRd 12 cycles (arm C)	Induction (cycles 1–4) Carfilzomib 36 mg/m^2^ D1, 2, 8, 9, 15, and 16 of a 28-day cycle; lenalidomide p.o. 25 mg D1–21 Dexamethasone 20 mg D1, 2, 8, 9, 15, and 16) or Cyclophosphamide 300 mg/m^2^ on D1, 8, and 15 Intensification ASCT/mel200 or 4 KRd cyles (cycles 4–8) Consolidation (cycles 8–12) 4 cycles same as induction Maintenance KR vs. R (random)	474	Pre-maintenance response rates: ≥VGPR: 76% Arm A 89% Arm B 87% Arm C sCR: 32% Arm A 44 % Arm B 43 % Arm C Pre-maintenance MRD neg MFC 42% Arm A 58% Arm B 54% Arm C	NA	Dermatologic 1–13% Neutropenia 15–20% Thrombocytopenia 8–15% Infections 12–14% Increased liver enzymes 1–10% Hypertension 3–8% Cardiac 2–3%
NCT02874742 GRIFFIN [76]	Phase II	Dara-VRd induction Mobilization ASCT Dara-VRd consolidation Dara-R maintenance	Induction (cycles 1–6) Lenalidomide 25 mg p.o. D1–14 Bortezomib 1.3 mg/m^2^ sc D1, 4, 8, and 11 Dexamethasone 40 mg weekly Daratumumab 16 mg/kg iv D1, 8, and 15 of cycles 1–4 and on D1 of cycles 5–6. Maintenance (C7–32) lenalidomide 10 mg p.o. daily (15 mg beginning at cycle 10 if tolerated) on D1–21 every 28 days and Dara 16 mg/kg iv every 56 days; Maintenance lenalidomide may be continued beyond C32	207 (safety run in 16 pts)	Post-consolidation (Dara–VRd) 91% ≥VGPR 52% ≥CR of which 59% MRD neg vs. (VRd) 73% ≥VGPR 42% ≥CR of which 24% MRD neg	NA (FU 13.5 months)	>10% Cytopenia similar in two arms 40% of Dara infusion-related reaction (mainly G1–2)
NCT02541383 CASSIOPEIA [77]	Phase III	Dara-VTd-ASCT-Dara-VTd vs. VTd-ASCT-VTd Maintenance Daratumumab vs. observation	Induction (cycles 1–4) and consolidation (cycle 5–6) Bortezomib 1.3 mg/m^2^ D1, 4, 8, 11 Thalidomide 100 mg daily p.o. in all cycles Dexamethasone 40 mg p.o. or iv D1, 2, 8, 9, 15, 16, 22, and 23 of induction cycles 1 and 2 and D1 and 2 of induction cycles 3 and 4 and 20 mg on D8, 9, 15, 16 of induction cycles 3–4 and D1, 2, 8, 9, 15, and 16 of both consolidation cycles. Daratumumab iv 16 mg/kg once weekly in induction cycles 1 and 2 and once every 2 weeks during induction cycles 3 and 4 and consolidation Stem-cell mobilization with cyclophosphamide (3 g/m^2^) ASCT Mel200 mg/m^2^ iv conditioning Maintenance Daratumumab (16 mg/kg) every 8 weeks until disease progression or for a maximum of 2 years vs. observation	1085	Post-consolidation (Dara–VTd) 83% ≥VGPR 10% CR 28.9% sCR 64% MRD neg (MFC) 39% MRD neg (NGS) vs. (VTd) 78% ≥VGPR 6% CR 20.3% sCR 44% MRD neg (MFC) 23% MRD neg (NGS)	Probability: 18 months PFS (Dara–VTd) 93% vs. (VTd) 85% OS NR	Neutropenia 28% vs. 15% Thrombocytopenia 11 vs. 7% Neuropathy 9% vs. 9% GIT 16% Reaction infusion 4% in Dara–VTd
NCT01998971 MMY1001 [11]	Phase Ib	Dara-KRd	Daratumumab 16 mg/kg QW for cycles 1–2, Q2W for cycles 3-6, and Q4W (1st dose of Dara split over 2 days) Carfilzomib 20/36 mg/m^2^ iv weekly D1, 8 and 15 of each 28-day cycle (20 mg/m^2^ on cycle 1, D1) for ≤13 cycles or elective discontinuation for ASCT Lenalidomide p.o. 25 mg D1–21 and Dexamethasone 20–40 mg per week	22	Best response 33% VGPR 14% CR 43% sCR	1-year PFS 95%	Lymphopenia 50% Neutropenia 23% 1 (5%) cardiac grade 3 TEAE was observed (congestive heart failure)
NCT01217957 C16005 [78]	Phase I/II	Ixa-Rd induction ASCT ± Ixa maintenance	Induction 28-day cycles (cycles 1–12) Ixazomib p.o. 4 mg D1, 8, and 15 Lenalidomide p.o. 25 mg D1–21 Dexamethasone p.o. 40 mg D1, 8, 15, and 22 Discontinuation → ASCT Maintenance Only for patients who did not proceed to ASCT: Single-agent ixazomib, given at the last tolerated dose during induction	65	63% ≥VGPR 32% CR MRD neg 12.5% (MFC)	Median PFS 35.4 months	Neutropenia 14% Thrombocytopenia 9% GIT 6%

NDMM, newly diagnosed multiple myeloma; pts, patients; V, bortezomib; R, lenalidomide; d, dex, dexamethasone; C, cyclophosphamide; K, carfilzomib; T, thalidomide; iv, intravenous; D, day; ASCT, autologous stem-cell transplantation; Mel200, melphalan at 200 mg/m^2^; Bu, busulfan; Ixa, ixazomib; p.o., orally; G-CSF, granulocyte colony-stimulating factor; Dara, daratumumab; Pts, patients; PR, partial response; VGPR, very good PR; CR, complete response; sCR, stringent CR; MRD, minimal residual disease; MFC; multiparameter flow cytometry NGS, next-generation sequencing; N, number; neg, negative; TTP, time to progression; PFS, progression-free survival; OS; overall survival; FU, follow-up; mod, modified; NA; not available; NR, not reached; G, grade AE, adverse event; TEAE; treatment-emergent AE; GIT, gastrointestinal toxicity. QW, given every week; Q2W, given every two weeks; Q4W, given every 4 weeks.

**Table 5:** Newly diagnosed multiple myeloma: selected clinical trials enrolling transplant-ineligible patients.

Protocol	Phase	Treatment		Subjects	Response	TTP/PFS/OS	Toxicity (≥G3)
NCT00644228 SWOG S0777 [108]	Phase III	VRd vs. Rd	VRd 21-day cycles (cycles 1–8) Bortezomib 1.3 mg/m^2^ iv D1, 4, 8, and 11 +; Lenalidomide p.o. 25 mg daily D1–14 Dexamethasone p.o. 20 mg daily D1, 2, 4, 5, 8, 9, 11, and 12 Rd 28-day cycles (cycles 1–6) Lenalidomide p.o. 25 mg D1–21 Dexamethasone p.o. 40 mg D1, 8, 15, and 22	525	≥VGPR 43% vs. 32% CR 15.7% vs. 8.4%	Median PFS 43 vs/ 30 months, median OS 75 vs. 64 months	Neurological AEs 33% vs. 11%
NCT02195479 ALCYONE [27]	Phase III	Dara-VMp vs. VMp	Induction 42-day cycles (cycles 1–9) Bortezomib 1.3 mg/m^2^ sc twice-weekly on weeks 1, 2, 4, and 5 of cycle 1 and once weekly on weeks 1, 2, 4, and 5 of cycles 2–9) Melphalan p.o. 9 mg/m^2^ once-daily on D1–4 Prednisone p.o. 60 mg/m^2^, once-daily on D1–4 Daratumumab iv 16 mg/kg Dexamethasone 20 mg once-weekly in cycle 1, every 3 weeks in cycles 2–9, and every 4 weeks thereafter until disease progression	706	≥VGPR 71% vs. 49% ≥CR 42% vs. 24% MRD neg by NGS Dara-VMp arm: 22.3% VMp arm: 6.2%	Median PFS NR	Neutropenia 40% vs. 38% Anemia 16% vs. 19%, Thrombocytopenia 34% vs. 37% Pneumonia 11% vs. 4%
NCT02252172 MAIA [110]	Phase III	Dara-Rd vs. Rd	Dara–Rd 28-day cycles Daratumumab iv 16 mg /kg once-weekly during cycles 1–2, every 2 weeks during cycles 3–6, and every 4 weeks thereafter Lenalidomide p.o. 25 mg D1–21 Dexamethasone p.o. 40 mg D1, 8, 15, and 22 Rd 28-day cycle Lenalidomide p.o. 25 mg D1–21 Dexamethasone p.o. 40 mg D1, 8, 15, and 22	737	≥VGPR 79% vs. 53% ≥CR 47% vs. 24% MRD neg by NGS Dara–Rd arm: 24.2% Rd arm: 7.3%	Median PFS NR	Neutropenia 50% vs. 35% Anemia 12% vs. 20% Lymphopenia 15% vs. 11% Pneumonia 14% vs. 8%
NCT02513186 SARVRD [111]	Phase I	Isa-VRd induction + Isa-Rd maintenance (16)	Induction 6-week cycles Isatuximab iv 10 mg/kg (cycles 1–4) D1, 8, 15, 22, 29 (cycle 1),; D1, 15, 29 (cycles 2–32 (cycles 1–4) Lenalidomide p.o. 25 mg/day D1–14 and D22–35 (cycles 1–4) Dexamethasone 20 mg D1, 2, 4, 5, 8, 9, 11, 12, 15, 22, 23, 25, 26, 29, 30, 32, 33 Maintenance 4-week cycles Isatuximab iv 10 mg/kg on D1, 15 (all cycles) Lenalidomide p.o. 25 mg D1–21 (all cycles) Dexamethasone p.o. 40 mg D1, 8, 15, 22 (all cycles)	22	ORR 93% MRD neg by NGS 50% (33% at 10^−6^) NGF 44% (18% at 10^−6^)	NA	G≥3 AEs were reported in 10 (46%) and SAEs in 4 (18%) pts Lymphopenia (8/22) Neutropenia (4/22) Thrombocytopenia (4/22)

V, bortezomib; R, lenalidomide; d, dexamethasone; Dara, daratumumab; M, melphalan; p, prednisone; Isa, isatuximab; D, day; iv, intravenous; p.o., orally; sc, subcutaneous; PR, partial response; VGPR, very good PR; CR, complete response; MRD, minimal residual disease; neg, negative; NGS, next-generation sequencing; NGF, next-generation flow; G, grade; AEs; adverse events; SAEs, serious AEs.

## Abbreviations

IMWG	International Myeloma Working Group
MRD	minimal residual disease
NGF	next-generation flow
NGS	next-generation sequencing
FLC	free light chain
M-protein	myeloma protein
FCM	flow cytometry
SUVmax	maximum standardized uptake value
MFC	multiparameter flow cytometry
FDG	Fluorodeoxyglucose
PET/CT	Positron emission tomography/computed tomography
N	number
TTP	time to progression
y	year
NR	not reached
CA	chromosomal abnormalities
PCs	plasma cells
BMPCs	bone marrow PCs
HR	hazard ratio
FLCr	free light chain ratio
FISH	fluorescence in situ hybridization
Pts	patients
V	bortezomib
R, Len	lenalidomide
d, dex	dexamethasone
T	thalidomide
FU	follow-up
PFS	progression-free survival
OS	overall survival
G	grade
P	*p*-value
MM	multiple myeloma
K	carfilzomib
CRAB	hypercalcemia, renal failure, anemia, and bone lesions
SMM	smoldering MM
ORR	overall response rate
Dara	daratumumab
ASCT	autologous stem-cell transplantation
Y	years
NA	not available
iv	intravenous
D	day
AE	adverse event
sc	subcutaneous
p.o.	orally
G-CSF	granulocyte colony-stimulating factor
Obs.	observation
TE	Transplant-eligible
NTE	non-transplant-eligible
NDMM	newly diagnosed multiple myeloma
C	cyclophosphamide
PD	progressive disease
MR	minimal response
PR	partial response
VGPR	very good PR
CR	complete response
nCR	near CR
sCR	stringent CR
CHF	congestive heart failure
SPMs	second primary malignancies
MRD neg	MRD negative/negativity
M, Mel	melphalan
Mel200	melphalan at 200 mg/m^2^
Bu	busulfan
p	prednisone
Ixa	ixazomib
SAEs	serious AEs
TEAEs	treatment-emergent AEs
GIT	gastrointestinal toxicity
QW	given every week
Q2W	given every two weeks
Q4W	given every 4 weeks
CI	confidence interval
ISS	International Staging System
R-ISS	Revised ISS
LDH	lactate dehydrogenase
EMD	extramedullary disease
CPC	circulating plasma cells
mAb	monoclonal antibody
PI	proteasome inhibitor
IMiDs	immunomodulatory drugs
